# Assessment of the operation status of electronic prescription at community pharmacies in Chengdu, China: a simulated patient study

**DOI:** 10.1186/s12913-023-09742-8

**Published:** 2023-08-29

**Authors:** Wenxin Zhou, Jing Wang, Qinmin Chen, Zhen Huang, Naitong Zhou, Ming Hu

**Affiliations:** 1 School of Pharmacy, Sichuan University, No. 17, 3Rd Section, Renmin South Road, 610041 Chengdu, P.R. China; 2 Clinical Research Institute, the Affiliated Hospital of Southwest Medical University, 646000 Luzhou, P.R. China; 3 Market Supervision Administration of Chengdu Municipality, 610041 Chengdu, P.R. China

**Keywords:** Community pharmacies, Electronic prescription, Policy, Simulated patient, Telemedicine

## Abstract

**Background:**

Information and technologies relevant to eHealth have developed rapidly over the past two decades. Based on this, China piloted "Internet + " pattern and some regions piloted electronic prescription services to explore telepharmacy services.

**Objective:**

To describe the processes and assess the operation status of electronic prescription services mode for community pharmacies in China.

**Methods:**

The simulated patient methodology was used to conduct a cross-sectional study in 317 community pharmacies from six districts in Chengdu, China in 2019. Simulated patients expressed three levels of service demands based on scenario about acute upper respiratory tract infections to evaluate the recommendation strength of electronic prescription services and telepharmacy service in community pharmacies. The descriptive statistics was completed to obtain the characteristics of the visit process, student t-test and χ^2^ test (*P* < 0.05 was considered statistically significant) were used for inferential statistical analysis to determine differences in characteristics and degree of recommendation between pharmacies.

**Results:**

Three Hundred Seventeen record sheets were effectively collected. The third-party platform was recommended in 195 (61.5%) interactions. The main reason for not recommending is non-prescription dispensing of prescription drugs (27.1%). 90.3% interactions waited less than 1 min, the counseling duration was less than 5 min in all interactions, and most community pharmacies had good network conditions (81.5%). 97.4% remote physicians offered professional counseling, only 22.1% of the pharmacists provided medication advice.

**Conclusions:**

The electronic prescription services mode for community pharmacies in Chengdu provides a convenient drug purchase process but remains some problems. For example, prescribing drugs without a prescription and services provided by pharmacists was poor, etc. The relevant supporting policies should be improved in future development process.

**Supplementary Information:**

The online version contains supplementary material available at 10.1186/s12913-023-09742-8.

## Background

Remarkable information and communication technologies progress have occurred over the past two decades, and eHealth has been the focus of the world. In 2005 World Health Assembly, the World Health Organization (WHO) encouraged member States to incorporate eHealth into public health so as to support for universal health coverage [1]. Under this background, the number of countries adopting eHealth policies or strategies increased substantially [2]. In a broad sense, eHealth is to optimize information delivery through electronic means to manage healthcare systems and support healthcare services [2]. The key fields of eHealth include electronic health record (EHR), electronic prescription, telemedicine, electronic identifiers, etc. [3]. Research showed that eHealth improved clinical efficacy and contributed to public health outcomes [4–7].

China adopted the eHealth strategy in 2012 and piloted "Internet + " pattern in some regions gradually [2]. In 2016, Chinese government began to encourage qualified community pharmacies to undertake medical institutions' outpatient pharmacy services and other professional services and explore innovation service patterns. In order to build a safe medication platform, the Administration for Market Regulation of Chengdu took the lead in exploring telepharmacy service in 2013 and piloted electronic prescription services in 2016, and initially formed the electronic prescription services mode (EPSM) for community pharmacies (including telepharmacy services and electronic prescription services).

**Fig. 1:**
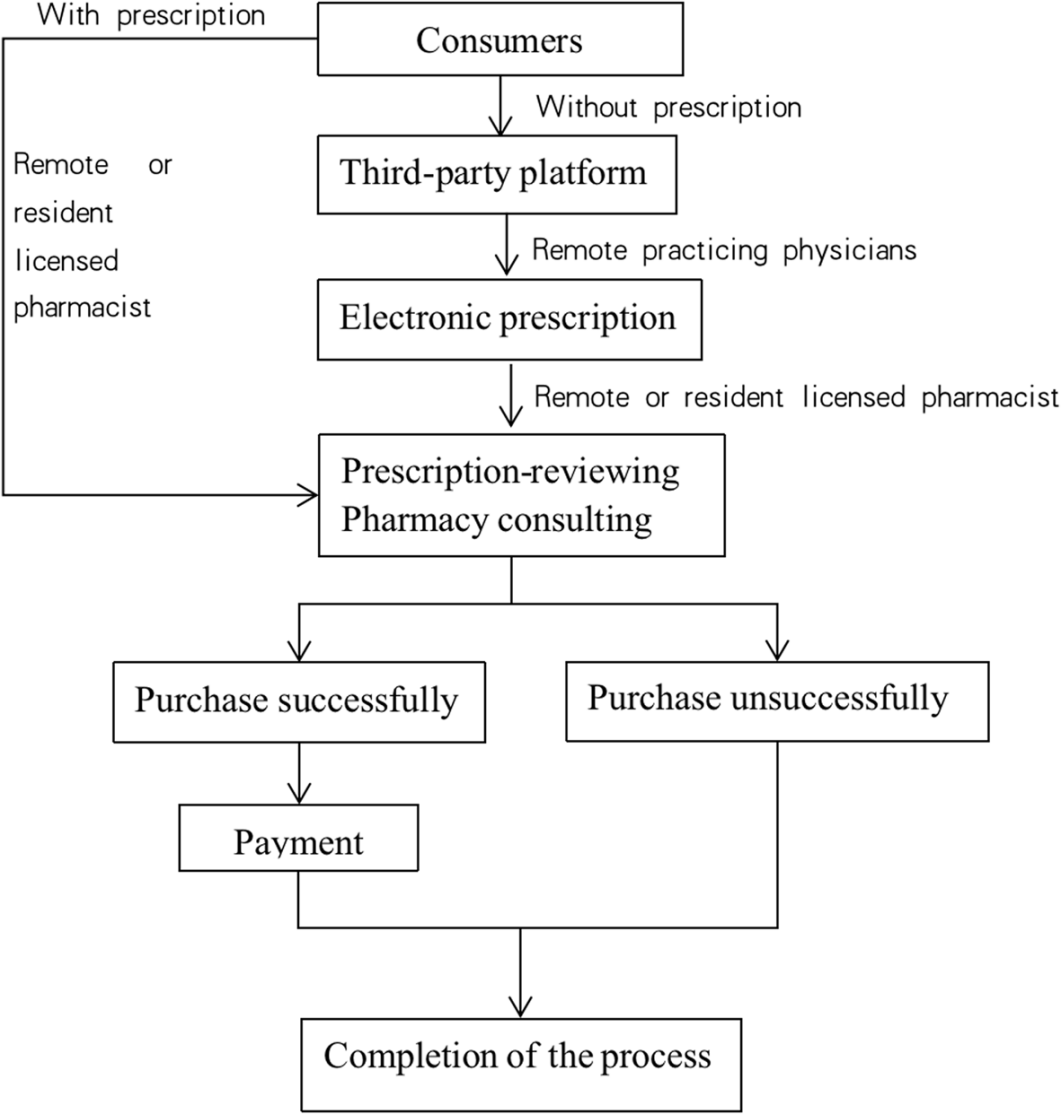
The process of EPSM of community pharmacies

In this pattern, community pharmacies cooperate directly with a legally qualified third-party platform, which signed medical institutions, physicians, and licensed pharmacists. If consumers bring their prescriptions when purchasing prescription drugs, the remote or resident licensed pharmacist can provide prescription review and rational medication guidance services. If not, the pharmacy staff will guide consumers to use a third-party platform or a remote platform, and the remote physicians can provide online counseling service and prescribe medication through the internet information system (electronic prescription services). After that, the pharmacist will dispense drugs and consumers can complete the purchase process (Fig. 1).

The sources of physicians from the third-party platforms include: licensed physicians from medical institutions, such as hospitals, community health service centers or township health centers and full-time licensed physicians of the platform. The sources of pharmacists who provide remote services include: remote licensed pharmacists in the headquarters of chain enterprises, resident licensed pharmacist and independent licensed pharmacist on third-party platforms. Under the EPSM mode, the third-party platforms do not charge consumers any service fees, but the corresponding service fees paid by community pharmacies to third-party platforms. The third-party platform uses technical means such as electronic signature of licensed physicians and cloud server to save electronic prescriptions, which can be used as evidence for investigation and evaluation of possible missed diagnosis, misdiagnosis, improper use of drugs or medication errors. In the meantime, the government will also conduct regulation on the telepharmacy and electronic prescriptions of community pharmacies involved in the drug retail process through flight inspections and other means.

By the end of December 2018, 9 third-party platforms were set in Chengdu, which provided telepharmacy service and electronic prescription service for over 6000 community pharmacies and 13000000 consumers.

The EPSM mode helps to solve the problems of prescription drug sales and supervision in community pharmacies, and facilitates the purchase of drugs by patients and the sharing and reasonable circulation of prescription information. Currently, the clinical, economic and human outcomes of electronic prescription service and telepharmacy service have been mentioned in several studies, including improving medication therapy outcomes, reducing the risk of medication errors and adverse drug events, reducing the expenditure of health care, facilitating the purchasing of drugs, benefiting the patient's adherence and satisfaction [8–13]. However, the operation status of this pattern are unclear, and more details about the the uses of the third-party platforms and the services of telepharmacies remains to be explored.

## Methods

### Ethics approval

Ethics for this study was approved by the Ethics Committee for Medical Research of Sichuan University (reference No. K2019034), a waiver of informed consent from pharmacies by the committee was obtained.

### Pharmacy selection

The online Raosoft calculator was used to calculate the sample size [14]. According to the 50% response distribution (out of 6000 pharmacies), 6% margin of error and 95% confidence level, the calculated sample size was 256. According to the calculation results, the final sample size was extended to 300 pharmacies. The study was conducted from March to April 2019 in the city of Chengdu, Sichuan province. Multi-stage sampling method was adopted for the selection of sample community pharmacies. First, six districts(Wuhou, Qingyang, Pidu, Shuangliu, Jianyang, Dayi) were selected from 20 districts in Chengdu according to the geographical location: two districts were selected respectively from main urban, suburban, and outer suburban. Then, according to the data of pharmacies that have carried out EPSM pilot in the database of Chengdu Municipal Drug Administration, 50 pharmacies were randomly selected from each of the six districts, a total of 300 sample pharmacies. To obtain sufficient valid data, we increased the sample size by 5%-10%. Each pharmacy was visited once using standard scenarios, expecting to conduct 315 to 330 visits.

### Simulated patients

Simulated patient method was adopted in this study. In this method, simulated patients were trained strictly and were indistinguishable from real patients. The simulated patients evaluated the services provided by community pharmacies through preset processes and scenarios. 10 graduate students of pharmacy from Sichuan University were recruited as simulated patients (aged 20- 25 years), in groups of 2 each. To ensure the survey was conducted consistently, simulated patients attended centralized training and rehearsed as the standard scenario before formal surveys. Then, each group selected three community pharmacies for pre-survey to familiarize themselves with the survey process and record sheet. During the formal survey, two simulated patients visited the pharmacy simultaneously, with one who portrayed acute upper respiratory tract infections. After the survey, the two simulated patients left together and wrote the record sheets within 15 min. The content of the record sheet included five parts: basic information of the pharmacy, sales of prescription drugs, network configuration, recommendation level of the third-party platforms and the implementation of electronic prescriptions.

### Scenarios

Electronic prescriptions are mainly applied in common and chronic diseases, with a fixed dosage. The reason for choosing Acute Upper Respiratory Tract Infections (AURTI) as a simulation scenario is that AURTI are the most common diagnoses in primary care [15], and adults suffer 2–4 URTI every year [16].

To evaluate the recommendation degree of electronic prescriptions services and telepharmacy service in community pharmacies, simulated patients expressed three levels of service demands according to the process and scenario set in advance:

1. "stating symptoms" (level 1)
2. "requesting prescription drugs" (level 2)
3. "requesting counseling a physician" (level 3)

**Fig. 2:**
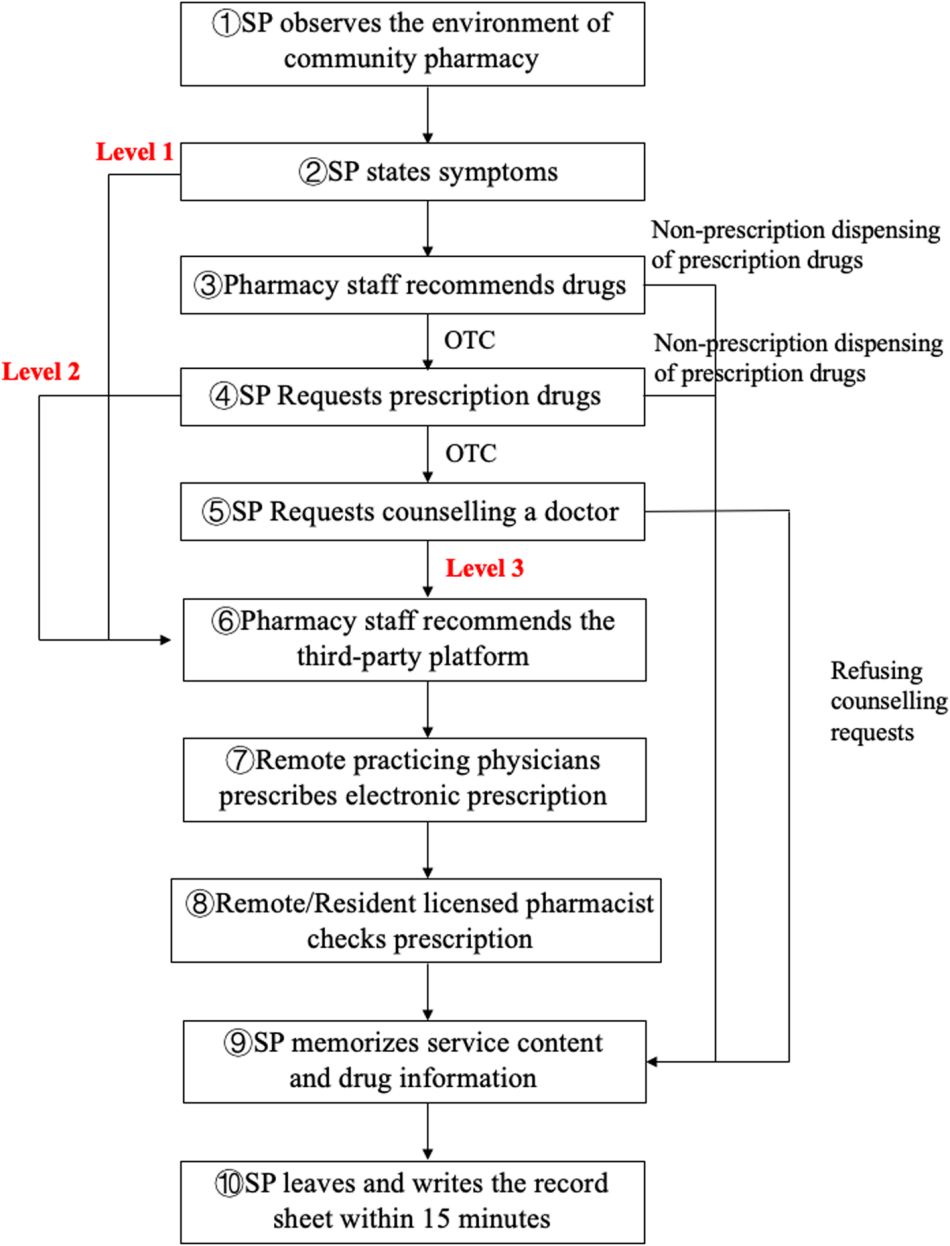
Flow chart of the purchase process

Pharmacy staff would recommend third-party platforms, dispense prescription drugs directly, or refuse counseling requests (Fig. 2).

### Statistical analysis

We sorted and numbered all record sheets, and excluded the invalid records. The descriptive statistics were conducted to demonstrate the characteristics of the visited pharmacies, the recommendation degree of third-party platforms, the hardware and network condition, and the service quality of remote physicians and pharmacists. Student t-test and χ^2^ test (*P* < 0.05 was considered statistically significant) in SPSS 26.0 statistical software were used for inferential statistical analysis to determine differences in characteristics and degree of recommendation between pharmacies.

## Results

### The completion of the survey

Three hundred twenty-eight community pharmacies were visited and 317 record sheets were effectively fulfilled, including 105 in main urban, 101 in suburban, and 111 in outer suburban. The response rate was 96.65%.

### The basic information of visited pharmacies

288 (90.9%) of 317 pharmacies were chain pharmacies, and 29 (9.1%) were independent pharmacies. The size of pharmacies varied significantly across different regions (χ^2^ = 13.773, *P* = 0.008) (Table 1).

**Table 1:** Characteristics of the visited pharmacies

Pharmacy characteristics	Main urban (*N* = 105)	Suburban (*N* = 101)	Outer suburban (*N* = 111)	Total (*N* = 317)
Type(χ^2^ = 2.564, *P* = 0.277)				
Chained pharmacy	98 (93.3%)	93 (92.1%)	97 (87.4%)	288 (90.9%)
Independent pharmacy	7 (6.7%)	8 (7.9%)	14 (12.6%)	29 (9.1%)
Size(χ^2^ = 13.773, *P* = 0.008)				
Large (> 100 m^2^)	20 (19.0%)	13 (12.9%)	33 (29.7%)	66 (20.8%)
Middle(50 ~ 100 m^2^)	61 (58.1%)	71 (70.3%)	52 (46.9%)	184 (58.0%)
Small (< 50 m^2^)	24 (22.9%)	17 (16.8%)	26 (23.4%)	67 (21.1%)
Had a particular zone of eHealth services(χ^2^ = 2.524, *P* = 0.281)				
Yes	93 (88.6%)	92 (91.1%)	105 (94.6%)	290 (91.5%)
No	12 (11.4%)	9 (8.9%)	6 (5.4%)	27 (8.5%)

A particular zone for electronic prescription and telepharmacy services was available at 290 (91.5%) pharmacies. 112(57.4%) pharmacies were equipped with the video camera, microphones, prescription camera (a camera specially used for taking pictures of prescriptions) and computers at the same time. Most community pharmacies had good network conditions (81.5%). A few simulated patients reported problems such as video streaming was slow buffering, voice intercom was not fluent and slow network speed.

### Degree of recommendation for pharmacies to use the third-party platforms

The third-party platforms were recommended in 195 (61.5%) interactions, but the recommendation rate varied across regions and the difference was statistically significant (χ^2^ = 10.902, *P* = 0.004). Only 26 (13.3%) interactions recommended third-party platforms when simulated patients stated symptoms (level 1). Most third-party platforms were recommended when simulated patients requested prescription drugs: 147(75.4%) (level 2). 22 (11.3%) interactions recommended when simulated patients requested counseling physicians (level 3). Although the recommendation rate was significantly different(χ^2^ = 11.364, *P* = 0.003), each region had the same characteristics in total——the level 2 had the largest proportion. The reasons for not using the third-party platform could be divided into three aspects: the prescription drugs were sold directly to patients, pharmacy staff refused to provide the third-party platform, and the third-party platform was not available (Table 2). Among the 86 interactions of pharmacy staff selling prescription drugs directly to patients, half of the interactions were pharmacy staff recommending prescription drugs and dispensing without prescription, the other half were sold right after the simulated patients asked for prescription drugs.

**Table 2:** The recommendation condition of third-party platform in community pharmacies

Pharmacy characteristics	Main urban(*N* = 105)	Suburban(*N* = 101)	Outer suburban(*N* = 111)	Total (*N* = 317)
Recommended third-party platform(χ^2^ = 10.902, *P* = 0.004)				
Yes	73 (69.5%)	49 (48.5%)	73 (65.8%)	195 (61.5%)
No	32 (30.5%)	52 (51.5%)	38 (34.2%)	122 (38.5%)
Recommendation level(z = 11.364, *p* = 0.003)				
Level 1: stating symptoms	6 (5.7%)	11 (10.9%)	9 (8.1%)	26 (8.2%)
Level 2: requesting prescription drugs	57 (54.3%)	34 (33.7%)	56 (50.5%)	147 (46.4%)
Level 3: requesting counseling physicians	10 (9.5%)	4 (4.0%)	8 (7.2%)	22 (6.9%)
The reasons for not using the third-party platform(χ^2^ = 5.486, *P* = 0.241)				
the prescription drugs were sold directly to patients	20 (19.0%)	38 (37.6%)	28 (25.2%)	86 (27.1%)
Pharmacy staff refused to provide third-party platform	5 (4.8%)	11 (10.9%)	5 (4.5%)	21 (6.6%)
The third-party platform was not available	7 (6.7%)	3 (3.0%)	5 (4.5%)	15 (4.7%)

When simulated patients used the third-party platform, 176(90.3%) pharmacies waited within 5 min, and the counseling duration was less than 5 min in all interactions.

**Fig. 3:**
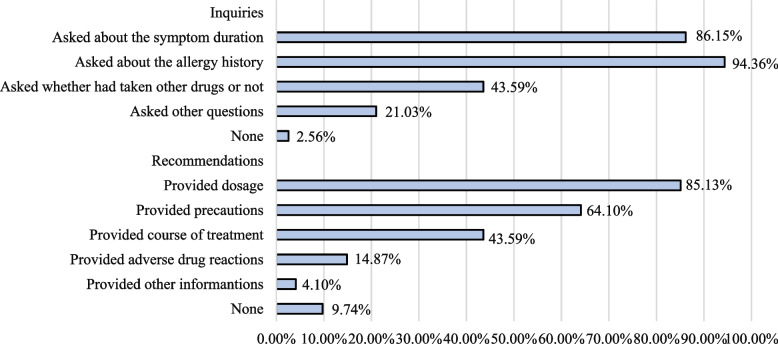
The counseling content of physicians' inquiries and recommendations

Figure 3 summarizes the results of counseling content of physicians' inquiries and recommendations. Physicians provided inquiries in almost all interactions (97.4%), inquiries about allergy history (94.4%) and symptom duration (81.2%) were the most common one. Besides, inquiries about taking other medicines were done in 85 (43.6%) cases. Recommendations were given by physicians in 176 (90.3%) out of 195 interactions, including the dosage of drugs (85.1%), precautions (such as not drinking after taking cephalosporin) (64.1%) etc. However, only 29(14.9%) physicians warned simulated patients about adverse drug reactions. More details about physicians' inquiries and recommendations are given in the appendix (pp 2).

After counseling, most electronic prescriptions(73.8%) were checked by remote licensed pharmacists through the internet information system, and the rest (26.2%) were reviewed by resident pharmacists or staffs. 18.5% of the pharmacists or staffs did not dispense following the electronic prescription, and only 22.1% of the pharmacists provided medication advice to simulated patients. More details about pharmacists' services are given in the appendix (pp 3).

## Discussion

Simulated patients can complete the purchase process according to standard scenarios, observe the actual responses of pharmacy staff in a natural environment. Compared with field survey and interview study, simulated patient study can reflect the actual situation of pharmacy service more truthfully [17]. This method has been used to assess the quality of pharmacy services and the accuracy of antibiotic dispensing. Furthermore, it can serve as an assessment and educational tool to enhance the quality of counseling [17–19]. It is the first time to use this method to evaluate the operation status and implementation effect of EPSM of community pharmacies, which provides valuable information for the implementation and development of the mode.

According to the Annual Statistical Report on Drug Regulation (2019), by the end of December 2019, China had 6701 chain pharmacy enterprises, 524000 community pharmacies (including 290000 chain pharmacies and 234000 independent pharmacies) [20]. Simultaneously, the number of licensed pharmacists registered in community pharmacies in China was 516000 [21]. This means that many community pharmacies do not have an adequate number of licensed pharmacists available. On the other hand, community pharmacies can not access the prescriptions because of the restriction of prescription outflowing by the medical institutions, which results in the phenomenon of non-prescription dispensing of prescription drugs. The EPSM of community pharmacies based on " Internet + Drug Circulation" has effectively solved the problem of limited sources of prescriptions, but the rationality of prescriptions needs to be further explored [22].

In this study, half of the non-prescription dispensing interactions happened because simulated patients asked for prescription drugs, demonstrating that pharmacy staff tend to meet patients' expectations of prescription drugs, regardless of whether it is appropriate or not [23–25]. 169 (86.7%) of 195 pharmacy staff recommended third-party platforms after simulated patients requested prescription drugs (level 1) or counseling physicians (level 2), indicating that patient s' needs play a vital role in the recommendation and use of third-party platforms. Besides, the proportion of level 2 is far more than level 1, explaining that the third-party platform is used as a tool to meet regulations rather than a counseling platform.

The main reason 122 interactions didn’t recommend third-party platforms was because pharmacy staff dispensed prescription drugs (such as Amoxicillin, Ampicillin, Cephalosporin, Pithecellobium clypearia, etc.) without prescription (70.5%). Non-prescription access to antibiotics is widespread around the world. Thirty-five studies from five continents showed that the frequency of non-prescription dispensing varied from 3–58% [26]. A multi-center cross-sectional study showed that antibiotics were easily obtained without a prescription when claiming the symptoms of URTI (70·1%) [18]. Multiple studies in China have demonstrated that the rate of dispensing antibiotics without a prescription varies between 63.1% to 73.3% [26–28]. Non-prescription dispensing of antibiotics occurred in one-sixth of the current study interactions, indicating that EPSM of community pharmacies may play an important role in avoiding the illegal sale of antibiotics, but the specific data needs further research.

In this study, most remote physicians provided counseling services while pharmacists seldom provided relevant medication guidance. Similar studies which also used the simulated patient method revealed that the quality of inquiries provided by pharmacists was poor. Pharmacists dispensed antibiotics without asking patients' condition and allergies history [18, 29, 30], which may be caused by physicians and pharmacists' different functional position. In terms of recommended items, physicians and pharmacists have similar performances. The most commonly recommended item was about dosage, while the least commonly recommended item was about adverse reactions, indicating that attention to the safety of the treatment was insufficient. This result is analogous to other international studies [29–34]. Only about one-fifth of pharmacists provided information, which is considerably less than similar studies [29, 33]. We detected that remote physicians have already provided simulated patient with relevant information during the counseling, and the pharmacists may prefer not to provide same information repeatedly. Patients should feel encouraged to ask questions and seek further information if needed [30].

Furthermore, we observed pharmacy staff did not dispense drugs according to the electronic prescription in 18.5% interactions during our purchase process, the main reason is that the community pharmacy did not equip with these drugs. Specifically, each community pharmacy is equipped with different varieties and specifications of drugs, but physicians as a third party were not clear on the availability of the drugs in various community pharmacies. When physicians prescribed drugs which were not provided in community pharmacies, driven by the profits and patients' needs, pharmacy staff tended to dispense similar drugs to simulated patients.

There are no uniform standards for the remote practicing physician's equipment and remote counseling protocol at present. Moreover, related regulations and supervision mechanisms have not been established, and the quality of EPSM of community pharmacies varies considerably. We identified some situations which would pose risks to the EPSM during our purchase process. Firstly, pharmacy staff requested specific antibiotics from physicians directly without counseling, only to meet regulations. Secondly, there are no effective measures to verify the authenticity of simulated patients' identity information. Thirdly, the EPSM isn't allowed for infants, minors, pregnant women and people over the age of 65 theoretically, but it isn't strictly enforced in the practical environment.

This study has some limitations. First, compared with chronic diseases such as diabetes, hypertension or heart disease, the limitation of antibiotics is stricter in China. If chronic disease is selected as simulated symptoms, the results of the study may be varied. Ssecond, we did not collect the audio recordings of the simulated patients, and the omissions of memory may affect the accuracy of the results [33]. Third, the purchase process of real patients may differ from the expected, thus the results in real situations may be different.

## Conclusions

Chengdu took the lead in exploring EPSM of community pharmacies, which conforms to the development trend of "Internet + ". EPSM facilitates community pharmacies transforming from traditional onsite service to the combination of online and onsite, which meets the actual needs of drug circulation industry and patients. The EPSM in Chengdu provides a new way to solve the problem of prescription drugs regulation and licensed pharmacist allocation. Using simulated patient method to assess the operation status and implementation effect of EPSM of community pharmacies, we found that more than half interactions recommend the third-party platforms. But the relevant supporting policies should be improved in future development process.

### Supplementary Information

**Additional file 1.** Supplementary appendix.

## Data Availability

All data generated during this study are included in this published article and its supplementary information files.

## Abbreviations

AURTI: Acute upper respiratory tract infections
EHR: Electronic health record
EPSM: Electronic prescription services mode
OTC: Over the counter medication
SP: Simulated patient
WHO: World health organization
