# Supplementary material for: Assessment of the operation status of electronic prescription at community pharmacies in Chengdu, China: a simulated patient study
Source: BMC Health Serv Res. 2023 Aug 29;23:920. doi: 10.1186/s12913-023-09742-8 (PMC10466720; doi:10.1186/s12913-023-09742-8)
Supplement: Supplementary file 1 — Additional file 1. Supplementary appendix. [file 12913_2023_9742_MOESM1_ESM.docx]

SUPPLEMENTARY APPENDIX

Supplement to:

Assessment of the Operation Status of Electronic Prescription at Community Pharmacies in Chengdu, China: A simulated patient study

Wenxin Zhou, Qinmin Chen, Jing Wang, Zhen Huang, Naitong Zhou*，Ming Hu*

Contents

1 The counselling contents of physicians' inquiries and recommendations

2 The services on medication guidance of pharmacists

1. The counseling contents of physicians' inquiries and recommendations

Table S1: The counseling contents of physicians' inquiries and recommendations

| **Pharmacy characteristics** | **Main urban（N=73）** | **Suburban（N=49）** | **Outer suburban（N=73）** | **Total**  **（N=195）** |
| --- | --- | --- | --- | --- |
| Contents of remote physicians' consultation | | | | |
| Symptom duration | 62 | 46 | 60 | 168 |
| History of drug allergy | 71 | 47 | 66 | 184 |
| Taking other medicines or not | 33 | 19 | 33 | 85 |
| Age | 71 | 45 | 66 | 182 |
| Other | 4 | 8 | 29 | 41 |
| None | 2 | 0 | 3 | 5 |
| Contents of remote physicians' medication guidance | | | | |
| Dosage | 66 | 48 | 52 | 166 |
| Administration | 66 | 43 | 49 | 158 |
| Course of treatment | 34 | 19 | 32 | 85 |
| Adverse drug reactions | 14 | 5 | 10 | 29 |
| Precautions | 54 | 29 | 42 | 125 |
| Other | 1 | 2 | 5 | 8 |
| None | 5 | 1 | 13 | 19 |

1. The services on medication guidance of pharmacists

Table S2: The services on medication guidance of pharmacists

| **Pharmacy characteristics** | **Main urban（N=73）** | **Suburban（N=49）** | **Outer suburban（N=73）** | **Total**  **（N=195）** |
| --- | --- | --- | --- | --- |
| The type of pharmacists on electronic prescription review | | | | |
| remote licensed pharmacists | 68 | 15 | 61 | 144 |
| resident pharmacists | 5 | 34 | 12 | 51 |
| Whether the pharmacists dispenses according to the electronic prescription or not | | | | |
| Yes | 52 | 46 | 61 | 159 |
| No | 21 | 3 | 12 | 36 |
| Contents of resident pharmacists' medication guidance | | | | |
| Dosage | 2 | 31 | 7 | 40 |
| Administration | 2 | 30 | 5 | 37 |
| Course of treatment | 1 | 13 | 6 | 20 |
| Adverse drug reactions | 0 | 4 | 2 | 6 |
| Precautions | 1 | 15 | 4 | 20 |
| Other | 0 | 0 | 0 | 0 |
| None | 3 | 1 | 4 | 8 |
